# Identification and validation of a novel anti-virulent that binds to pyoverdine and inhibits its function

**DOI:** 10.1080/21505594.2020.1819144

**Published:** 2020-09-22

**Authors:** Xu Wang, Quinn Kleerekoper, Alexey V. Revtovich, Donghoon Kang, Natalia V. Kirienko

**Affiliations:** a Department of BioSciences, Rice University, Houston, TX, USA; b Shared Equipment Authority, Rice University, Houston, TX, USA

**Keywords:** *Pseudomonas aeruginosa (P. aeruginosa)*, pyoverdine, solution nuclear magnetic resonance (NMR), *in silico* structure modeling, structure-activity relationship (SAR)

## Abstract

*Pseudomonas aeruginosa*: causes serious infections in patients with compromised immune systems and exhibits resistance to multiple antibiotics. The rising threat of antimicrobial resistance means that new methods are necessary for treating microbial infections. We conducted a high-throughput screen for compounds that can quench the innate fluorescence of the chromophore region of the *P. aeruginosa* siderophore pyoverdine, a key virulence factor. Several hits were identified that effectively quench pyoverdine fluorescence, and two compounds considerably improved the survival of *Caenorhabditis elegans* when worms were challenged with *P. aeruginosa*. Commercially available analogs of the best hit, PQ3, were tested for their ability to rescue *C. elegans* from *P. aeruginosa* and to interact with pyoverdine via fluorescence and solution NMR spectroscopy. ^1^H-^15^N and ^1^H-^13^C HSQC NMR were used to identify the binding site of PQ3c. The structure model of pyoverdine in complex with PQ3c was obtained using molecular docking and molecular dynamics simulations. PQ3c occupied a shallow groove on pyoverdine formed by the chromophore and N-terminal residues of the peptide chain. Electrostatic interactions and π-orbital stacking drove stabilization of this binding. PQ3c may serve as a scaffold for the development of pyoverdine inhibitors with higher potency and specificity. The discovery of a small-molecule binding site on apo-pyoverdine with functional significance provides a new direction in the search of therapeutically effective reagent to treat *P. aeruginosa* infections.

**Abbreviations**: NMR: nuclear magnetic resonance; SAR: structure–activity relationship; MD: molecular dynamics; RMSF: root-mean-square fluctuation; HSQC: heteronuclear single quantum correlation; DMSO: dimethyl sulfoxide; Δδ_avg_: average amide chemical shift change; DSS: 2,2-dimethyl-2-silapentane-5-sulfonate; RMSD: root-mean-square deviation; LJ-SR: Lennard–Jones short-range; Coul-SR: Coulombic short-range; FRET: fluorescence resonance energy transfer

## Introduction

*Pseudomonas aeruginosa*, a Gram-negative bacterium and an opportunistic pathogen, is the major cause of morbidity and mortality in patients with cystic fibrosis, and is a serious threat for patients who are immunocompromised or have severe burns. It is also a leading cause of nosocomial infections, and frequently colonizes surgical sites, medical implants, and therapeutic machinery, like ventilators. The intrinsic and adaptive resistance of *Pseudomonas aeruginosa* to a wide variety of antimicrobials and disinfectants makes it difficult to eliminate from clinical settings, and its environmental ubiquity makes any such removal short-term. Compounding the problem, *P. aeruginosa* readily acquires new antimicrobial resistance mechanisms, resulting in multidrug-resistant or even pandrug-resistant strains that exhibit mortality rates as high as 70%[1]. Unfortunately, a timeline comparing antimicrobial discovery and the emergence of resistance tells a clear tale: bacteria develop resistance at a rate that is prodigiously faster than new classes of antimicrobials (or even new generations of current antimicrobials) can be identified. While discovery of these compounds remains an important goal, it should only be considered a stop-gap method while new treatment strategies are being developed.

One promising option may be to target bacterial virulence factors for therapeutic intervention [2–4]. In principle, disrupting key virulence factors could have a substantial effect on bacterial pathogenesis. Importantly, anti-virulents may have less impact on growth than conventional antimicrobials, which would reduce the evolutionary drive of the pathogen to acquire resistance [5]. As such, the clinical life of these treatments may be greatly extended when compared to classical antimicrobials. Recent evidence from multiple labs also suggests that anti-virulents and conventional antimicrobials may be combined into treatment “cocktails” that are more effective than either treatment alone [6–10].

Despite their promise, several significant obstacles have limited the development of anti-virulents. First, relevant targets need to be identified. For many pathogens, this step is surprisingly difficult, as the molecular determinants of microbial pathogenesis tend to be complex and multifactorial. However, it is not always clear that even fully inhibiting a single virulence factor will have a significant impact on pathogenesis; host–pathogen interactions are complex. But even if a target is identified, and its disruption has a dramatic effect on the course of disease, the target may not be amenable to screening efforts (e.g., it may be insoluble, membrane-bound, overly difficult to purify, or non-druggable). Finally, the process of screening the targeted virulence factor is often laborious. Typically, a derivatized version of the virulence factor must be generated and evaluated for function under infection and screening conditions.

**Figure 1.**
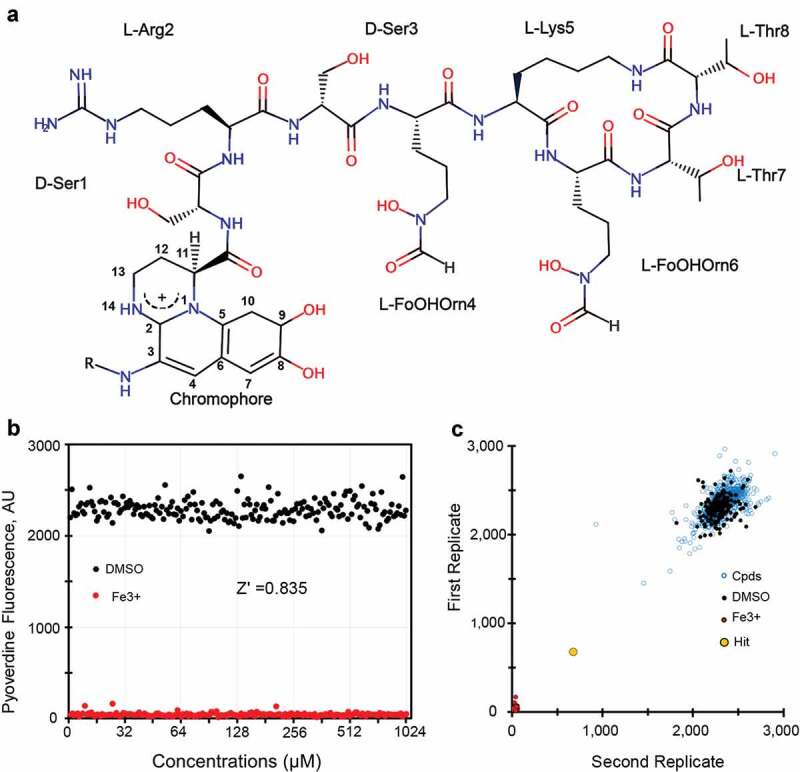
High throughput-screening for pyoverdine-inhibiting compounds. (a) Chemical structure of pyoverdine produced by *P. aeruginosa* PAO1. (b) Quantification of pyoverdine fluorescence in the presence of negative (DMSO) or positive control (ferric iron). (c) Representative data from four pooled screening plates, showing high correlation between replicates. X- and Y-axis show pyoverdine fluorescence (AFU) for respective replicates.

Pyoverdines are a class of closely-related but structurally diverse, mixed-type siderophores produced by fluorescent Pseudomonads that meet most of these criteria. All pyoverdines contain the same 2,3-diamino-6,7-dihydroxyquinoline-based chromophore. The catechol moiety at one end of the planar chromophore helps to coordinate the ferric iron, while the C-3 site is modified by the addition of a side chain composed of an α-ketoacid from the Krebs cycle (typically succinate, α-ketoglutarate, malate, or glutamate, or their monoamide derivatives). A mixture of the different acyl side chains are typically found. The carboxyl position of the chromophore is modified by an oligopeptide that often contains a variety of D-form and non-standard amino acids and is synthesized via non-ribosomal peptide synthesis. Three forms of pyoverdine (known as Type I, Type II, and Type III) are produced by Pseudomonads, with the most substantial differences appearing in their peptide chains [11]. Type I pyoverdines (pyoverdine I), whose representative pyoverdine is from *P. aeruginosa* strain PAO1 (Figure 1a), is often characterized by a peptidic part with the last three to four amino acids forming a cycle; type II pyoverdines (pyoverdine II), usually have a linear peptidic sequence with the last residue being an N-hydroxy(cyclo)Orn at the C-terminus; and type III pyoverdines (pyoverdine III), frequently have a linear peptidic sequence with an unmodified N-hydroxyOrn at the C-terminus. Interestingly, the structures for pyoverdine I from *P. aeruginosa* strain PAO1 and *P. aeruginosa* strain PA14 appear to be equivalent and are shown (Figure 1a). For the remainder of this manuscript, we will use pyoverdine to mean this structure, with varying acyl groups, unless otherwise specified.

Pyoverdine is a key virulence determinant in hosts ranging from cultured cells to invertebrates to mammals [12–18]. It directly removes iron from hosts and disrupts their mitochondrial function [14,15,19] and also regulates the transcription of several important virulence factors, including the translational inhibitor exotoxin A, the protease PrpL, and pyoverdine itself [20–23]. Pyoverdine-mediated iron acquisition also facilitates biofilm production [24,25]. Importantly, all of these activities depend on pyoverdine’s ability to bind iron; if this ability is disrupted, pathogen virulence would be severely compromised.

In addition, pyoverdine is druggable; there have been multiple reports describing small molecules targeting either pyoverdine itself or its biosynthesis [9,12,26–32]. As it is a siderophore, pyoverdine is naturally secreted from bacteria at high levels and it is readily soluble in water. Usefully, the chromophore core confers both a visible color and a characteristic fluorescence (Ex_405_, Em_460_) that allow pyoverdine to be easily tracked for purification and analysis. Importantly, this fluorescence is rapidly and almost completely quenched when pyoverdine binds Fe^3+^.

In this study, we report a high-throughput biochemical screen to identify compounds that inhibit pyoverdine fluorescence, which serves as a proxy for its ability to chelate ferric iron. After hits were obtained, initial characterization and optimization was performed, leading to the identification of the small molecule PQ3 as a potential lead. A survey of commercially available analogs led to the identification of an analog, which we have named PQ3c, as a more effective anti-pyoverdine agent. Using NMR and molecular dynamics (MD) simulations, we identified a site on pyoverdine where PQ3c binds. Computational techniques were used to optimize the structures of the best complex from docking, to characterize the intermolecular interaction, and to provide insights on the molecular recognition mechanism. Electrostatic interactions between the peptide chain of pyoverdine and the compound and π orbital stacking appear to be the major driving forces for binding. These results provide a starting point for the discovery of high binding affinity pyoverdine inhibitors that may help treat *P. aeruginosa* infections.

## Materials and methods

### Strains

The temperature-sterile *C. elegans* strain SS104 *[glp-4(bn2)]* strain was used for all experiments. *C. elegans* were maintained on nematode growth medium (NGM) seeded with *E. coli* strain OP50 at 15°C [33]. For Liquid-Killing experiments (see below) either wild-type *P. aeruginosa* strain PA14, a clinical isolate described elsewhere [34] or PA14*pvdF* or PA14*pvdP* mutants with a Mariner transposon inserted into the *pvdF* or *pvdP* loci [35], respectively, were used. Transposon mutants were sequence-confirmed. Pyoverdine for NMR experiments was purified (see below) from *P. aeruginosa* strain PAO1. *E. coli* strain OP50 was used for chronic compound toxicity assays in *C. elegans*.

### Biochemical screen

Z’ factor determination for the pyoverdine fluorescence quenching screen was performed using black, clear-bottom 384-well plates. Each well contained 50 μL of partially purified pyoverdine in buffered media (pH = 6) at ~2,500–3,000 AFU/well. Pyoverdine concentration varied (within 500 AFU) from lot to lot, but each plate contained pyoverdine only from a single dilution. Half of the wells contained 50 μL DMSO as a negative control and half contained 50 μL of 100 µM iron (III) chloride as a positive control.

High-throughput chemical screening was performed at the Institute of Biotechnology – Texas A&M Health Science Center. 300 nL of compounds were pin transferred into duplicate plates at a final concentration of approximately ~20 µg/mL. Each 384-well plate contained at least 24 wells containing solvent controls and 4 wells containing iron (III) chloride.

### Compounds

Screening compounds were purchased from varying companies through the chemical marketplace MolPort (Riga, Latvia). The purity of each purchased compound was confirmed to be >90% by 1D and 2D ^1^H NMR. The tautomeric and ionization states of the compounds were predicted by MarvinSketch 19.1.0, 2019.

### Pyoverdine purification

For pyoverdine purification, *Pseudomonas aeruginosa* strain PAO1 was grown in iron-poor M9 media (1% (w/v) Difco 5X M9 salts, 11.3 g/L iron-limited casamino acids, 0.4% glucose, 1 mM CaCl_2_, 1 mM MgSO_4_) for 20–22 h at 37°C under shaking conditions. Bacteria were then pelleted by centrifugation at 10,000 g for 30 min and the culture supernatant was passed through a 0.22 μM filter twice. Pyoverdine was then purified in batch method from filtered supernatant by mixing with Amberlite XAD-4 resin and then and eluted with 50% v/v methanol. Eluent was dialyzed against Milli-Q H_2_O overnight and further purified by high-performance liquid chromatography on reverse-phase C18 preparative column with a 0–100% acetonitrile gradient at a flow rate of 5.0 ml/min.

Isotopically labeled (^15^N or ^13^C and ^15^N) pyoverdine was purified as above, except that *P. aeruginosa* PAO1 was inoculated in iron-poor M9 media supplemented with 1.0 g/L ^15^NH_4_Cl as the sole nitrogen and 2.0 g/L unlabeled or uniformly labeled ^13^C glucose as the carbon source for 20–22 h at 37°C. Labeled pyoverdine was then purified as described above.

### Fluorescence spectroscopy

Pyoverdine fluorescence was determined spectrophotometrically with excitation 405 nm and emission 460 nm using Cytation5 multimode plate reader (BioTek, VT) at room temperature. To determine the quenching effects of compounds on pyoverdine, the fluorescence of 10,000 AFU of pyoverdine was monitored in the presence of increasing concentrations of each compound (0, 1, 2, 4, 8, 16, 32, 64, 128, 256, 512, and 1024 μM). 12,000 AFU of pyoverdine correlates with approximately 10 µM pyoverdine at pH 6.0 in SK medium. Dissociation constants (*K*_d_) were derived from fluorescence by fitting titration curves to the following equation, which does not require measurement of free ligand concentrations [36,37].

(1) $$\Delta F=\Delta Fmax\frac{\begin{array}{rl} & \left(\left[P\right]t+\left[L\right]t+Kd\right)-\\ & \sqrt{(\left[P\right]t+\left[L\right]t+Kd-4\left[P\right]t\left[L\right]t} \end{array}}{2\left[P\right]t}$$

where *ΔF_obs_* is the change in the observed fluorescence intensity from the free state, *ΔF_max_* is the maximum fluorescence change, [*L*]_t_ is the total ligand added at a given titration point, [*P*]_t_ is the total pyoverdine concentration and *K_d_* is the dissociation constant.

### Liquid-Killing assay

The Liquid-Killing assay was performed as previously described [38,39]. In brief, 20 young adult *C. elegans* were deposited into each well of a black, clear-bottom 384-well plate. Liquid-Killing media (pH = 6), including *P. aeruginosa* at an OD_600_ of 0.03 was then added, and worms were incubated for ~40 h at 25°C. Afterward, worms were washed to remove bacteria, pyoverdine, and debris, and *C. elegans* were stained with the cell-impermeant fluorescent dye Sytox Orange (Invitrogen, CA) in minimal S Basal media. Quantification of dead worms in each well was assessed using Cell Profiler software (http://cellprofiler.org/).

### qRT-PCR

To assess the effect of pyoverdine inhibitors PQ3, PQ3c, and 5-FC on the expression of virulence factors whose transcription depends on PvdS, which is dependent upon ferripyoverdine binding FpvA, bacteria were grown for 12 h in SK media supplemented with either DMSO or 100 µM inhibitor. Then cells were collected, pelleted, and RNA was extracted and purified using TRIzol reagent (Invitrogen, CA, United States) according to the manufacturer’s protocols. To ensure cell lysis, cells were resuspended in TRIzol reagent and freeze-cracked and vortexed prior to phase separation. Purified RNA was treated with DNase I (Thermo Fisher Scientific, MA, United States). Reverse transcription was performed using random decamers and Retroscript Kit (Thermo Fisher Scientific, MA, United States). qRT-PCR was performed using SYBR green AzuraQuant Fast Green Fastmix (Azura, MA, United States) in a CFX-96 real-time thermocycler (Bio-Rad, CA, United States). Fold-changes were calculated using a ΔΔCt method. Primer sequences are available upon request.

### Mammalian cell viability assay

16HBE human bronchial epithelial cells were used to assess the toxicity of pyoverdine inhibitors. Cells were propagated in Eagle’s Minimum Essential Medium supplemented with 10% FBS, nonessential amino acids, and penicillin/streptomycin. During treatment, cells were exposed to pyoverdine inhibitors for 24 h. After that, to assess cell survival, alamarBlue cell viability agent was added. alamarBlue fluorescence was measured after 2 h of incubation.

### NMR methodology

All NMR experiments were carried out on a Bruker NEO 600 MHz NMR spectrometer equipped with a 5‐mm TXI H-C/N-D probe at 278 K. The NMR sample for backbone and side chain assignments contained 1.2 mM ^13^C, ^15^N pyoverdine I (purified from *P. aeruginosa* PAO1) in a buffer including 25 mM phosphate, 5% D_2_O, 5.0 µM 2,2‐dimethyl‐2‐silapentane‐5‐sulfonate (DSS), pH = 5.8. Backbone amide assignments were made using HNCO, HNCA, HN(CO)CA, HNCACB, CBCA(CO)NH and ^15^N-edited NOESY-HSQC experiments. Aliphatic side chain assignments were obtained using 2D ^1^H-^13^C HSQC, CCH-TOCSY, HCCH-TOCSY, and HCCH-COSY. The assignments of aromatic chromophore were made by^1^H-^13^C HSQC and ^13^C-edited NOESY-HSQC.

To determine the interactions between pyoverdine and each compound by NMR, 50 mM compound stock solutions were made in deuterated-dimethyl sulfoxide (d-DMSO). ^15^N or ^13^C HSQC spectra of 0.2 mM isotope-labeled pyoverdine in the presence of 5% (v/v) d-DMSO without compound was used as a background control, and the spectrum of pyoverdine in the presence of 2.5 mM compound was compared with the control to examine chemical shift changes. The average amide chemical shift change (Δδ_avg_) was calculated using the following equation [40]:

(2) $$\Delta \delta avg=\sqrt{\frac{{\left(\Delta \delta H\right)}^{2}+{\left(\Delta \delta N/5\right)}^{2}}{2}}$$

where ΔδH is the change in the ^1^H chemical shift, and ΔδN *i*s the change in the ^15^N chemical shift.

^1^H chemical shifts were referenced to DSS, and ^15^N and ^13^C chemical shifts were referenced indirectly using their respective gyromagnetic ratios [41]. NMR spectra were processed with nmrPipe [42] and analyzed with nmrView [43].

### Molecular docking

AutoDock Vina was used to predict compound binding [44,45]. Two conformations of apo-pyoverdine were used as target structures in docking experiments. The conformations were the representative structures of the two most populated clusters from apo-pyoverdine MD simulation. The chemical structure of PQ3c was sketched using MarvinSketch 19.1.0, 2019 and converted to a 3D structure using OpenBabel [46]. The partial charges of pyoverdine and PQ3c were assigned by the Gasteiger-Huckel method in AutoDock tools. The modified PDB files were converted to PDBQT format using AutoDock tools. The search space was defined to be a 3D grid box, 30 × 30 × 30 Å with a spacing of 0.375 Å, which encompasses the chromophore, D-Ser1, L-Arg2, and D-Ser3 from pyoverdine. Ten docking poses were obtained with an exhaustiveness value of 24 for each conformation. The complex of pyoverdine-PQ3c with the lowest docking scores was selected for the initial complex structure. The docking structures were illustrated and visualized using UCSF Chimera [47].

### Molecular dynamic simulations of apo-pyoverdine and its complex

All the MD simulations were performed using GROMACS version 2019.3 MD simulation package with CHARMM27 force field [48]. For the MD simulation of apo-pyoverdine, the starting structure was taken from the crystal structure of apo-pyoverdine complexed with its outer membrane transporter FpvA (PDB ID: 1xkh) [49]. The topology and parameter files for the nonconventional residues (chromophore and FoOHOrn) in pyoverdine were acquired from Swiss Sidechain database (https://www.swisssidechain.ch/). For the simulation of apo-pyoverdine in complex with PQ3c, the starting structure was taken from the top pose from the docking trial. The topology and parameter files of PQ3c were acquired from the Swiss Sidechain database.

All the systems were solvated in a triclinic water box using TIP3P water model (12.0 Å per side) and counter ions were added to neutralize the system based on the overall charge of the system. To remove improper atom contacts, the system was subjected to energy minimization on a convergence threshold of 1000 kJ mol^–1^ nm^–1^ using the steepest descent and then conjugated gradient minimization. Next, a two-step equilibration phase, constant volume (NVT) and constant pressure (NPT) was applied. Finally, the well-equilibrated systems were subjected to a production run at 300 K and 1 bar pressure for 50 ns with an integration time step of 0.2 ps using the leapfrog algorithm. Trajectory snapshots were stored at every 10 ps during the simulation period. The resultant trajectories were analyzed using GROMACS analysis tool kit. Trajectories were visualized by UCSF Chimera [47].

## Results

### High-throughput screen for pyoverdine-quenching compounds

In the course of our previous work, we made the serendipitous observation that several compounds that inhibit the activity of pyoverdine also quench its fluorescence [9]. We reasoned that this was likely to be due to interactions between the compound and pyoverdine, allowing non-radiative relaxation of the excited complex. In this case, the compound may compromise the coordination of the iron-chelating activity. On this basis, we decided to carry out a high-throughput, fluorescence-based screen for small molecules that may inhibit the function of pyoverdine.

Pyoverdine was partially purified from spent bacterial media by centrifugation and then filtration to remove bacterial cells. The resultant material was then autoclaved to denature the remaining proteins, followed by ultracentrifugation to remove as much insoluble material as possible. The resulting partially purified pyoverdine was measured via fluorescence spectroscopy, and then was diluted to a standard concentration (3,000 AFU) before being added to 384-well plates. Using iron (III) chloride and DMSO as positive and negative controls, the Z’ factor for the screen was established as 0.835, which corresponds to a difference of > 20 standard deviations (Figure 1b). This indicates that the screen has strong power to identify even very weak hits. ~45,000 wells from fragment-based, small molecule diversity libraries (including the Chembrige Diversity Set, Maybridge HitFinder (v9) Collection, NIH Custom Clinical collection, and the NCI Diversity Set III) were screened in duplicate (Figure 1c). Each plate contained iron (III) chloride and DMSO as positive and negative controls. Compounds were considered primary hits if they quenched pyoverdine fluorescence by > 50% at 40 μM.

Our biochemical screen resulted in the identification of 23 primary hits, 15 of which were commercially available. All fifteen compounds were purchased and solubilized in DMSO at a final concentration of 100 μM. Compounds were then rescreened against pyoverdine at a higher initial concentration (25,000 AFU) for the ability to quench pyoverdine fluorescence by > 60%. They were also counterscreened against 4-acetamido-4ʹ-isothiocyanato-2-2ʹ-stilbenedisulphonic acid (SITS), which has fluorescence spectra similar to pyoverdine, to eliminate compounds that do not quench pyoverdine fluorescence, but are acting as a FRET acceptor instead. Five compounds passed these counterscreens and were labeled PQ1-5 (Fig S1). Due to limited commercial availability and comparatively low solubility, PQ4 and PQ5 were triaged.

Based on previous results, we anticipated that these compounds would prevent pyoverdine function. To test this, compounds were examined for the ability to extend *C. elegans* survival during exposure to *P. aeruginosa* in a Liquid-Killing assay. In this assay, *C. elegans* are incubated in a microtiter plate with *P. aeruginosa* strain PA14. The low concentration of iron in the assay media triggers pyoverdine production, killing the worms after approximately 40 h. Since pyoverdine is required for killing in this time frame, compounds that prevent death are likely to limit siderophore function. Of the three molecules tested, PQ2 exhibited substantial toxicity and was deprioritized. PQ3 (5‐oxo-3‐phenyl‐4-[2‐(1,3‐thiazol-2‐yl)hydrazin-1‐ylidene]pyrazole-1‐carbothioamide) showed the best rescue and was selected for further analysis.

### Structure-activity relationship analysis of PQ3

**Figure 2.**
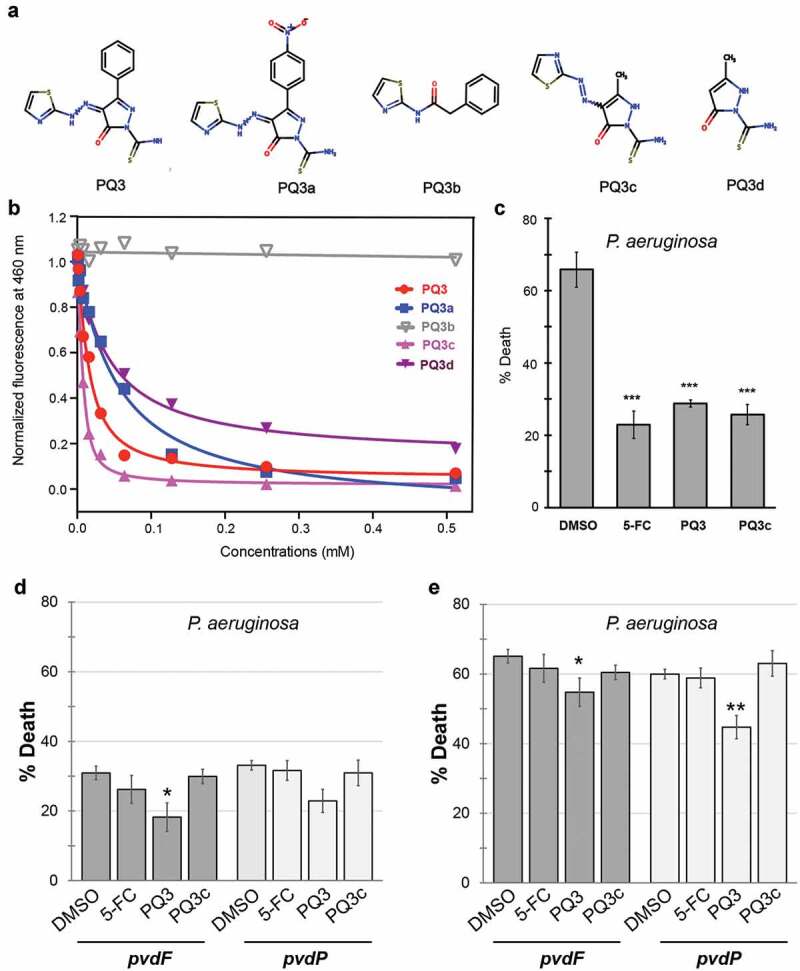
Characterization of anti-virulent properties of PQ3 and PQ3c. (a) Structure of PQ3 and its analogs. (b) Dosage-dependent effects of PQ3-family compounds on the innate pyoverdine fluorescence. (c-e) *C. elegans* survival after exposure to wild-type *P. aeruginosa* (c) or pyoverdine-deficient mutants (d-e) in the presence of PQ3 or PQ3c. DMSO and 5-fluorocytosine (5-FC) served as positive and negative controls, respectively. For (c-d), the same time point was used. For (e), a longer exposure was used in order to match the level of death between *C. elegans* exposed to wild-type *P. aeruginosa* or pyoverdine biosynthesis mutants. For (c-e), at least three independent biological replicates were performed. For each replicate, at least four wells with ~ 20 worms/well were used. Statistical significance was determined using Student’s *t*-test.

To investigate the relationship between chemical structure and pyoverdine binding, we searched for commercially available analogs of PQ3. We discovered a small number of analogs, each with a different substituent in the pyrazole ring. These compounds were subsequently named PQ3a, PQ3b, PQ3c, and PQ3d (Figure 2a).

As a first step, PQ3 and its analogs were tested for the ability to quench pyoverdine fluorescence in a dose-dependent fashion (Figure 2b). With the exception of PQ3b, which had no effect on pyoverdine fluorescence, each compound showed a dose-dependent response that fit well to a single-site binding model (see Equation (1) in Materials and Methods) [36,37]. Using these data, we calculated each molecule’s *K*_d_ for iron-free pyoverdine (Table 1).

**Table 1.** Dissociation constants (*K*_d_) for binding PQ3-family compounds to apo-pyoverdine.

Compound	*K*_d_ (µM)
PQ3	10.23 ± 1.80
PQ3a	50.61 ± 9.41
PQ3b	>1000
PQ3c	2.67 ± 0.42
PQ3d	39.5 ± 4.1

The parental compound PQ3 bound to pyoverdine with an affinity of *K*_d_ ≈10 μM. For PQ3a, replacement of the phenyl group by a bulkier nitrophenyl group at the C-3 position of the pyrazole ring reduced the affinity compared to PQ3. The lack of the negatively charged pyrazole ring likely prevented PQ3b from forming electrostatic interactions with the positively-charged ε-amine of L-Arg in the peptide chain of pyoverdine. The thiazole group alone appears to be insufficient to stabilize the interaction with the siderophore. The fact that no binding was observed between PQ3b and pyoverdine highlights the importance of the pyrazole ring in these intermolecular interactions. PQ3c, where the large aromatic phenyl was substituted with a smaller methyl group at position C-3 on pyrazole ring, had a significantly improved affinity (*K*_d_ = 2.67 μM), suggesting that the location of binding was sterically hindered from allowing the larger group. PQ3d, which has the same methyl group at C-3 as PQ3c, but no substituent at C-5, had comparatively lower affinity than either PQ3c or PQ3, indicating that this methyl moiety also contributes to binding stabilization.

Next, we examined the ability of PQ3c to function in a biological context. First, we tested the ability of PQ3c to alleviate pyoverdine-mediated pathogenesis in the *C. elegans* Liquid-Killing assay. PQ3 and PQ3c each exhibited significant rescue and were comparable to 5-fluorocytosine (5-FC, Figure 2c), a well-known inhibitor of pyoverdine biosynthesis [12,29].

To rule out the possibility that PQ3 and PQ3c were operating in some other fashion, we tested whether the compounds’ ability to rescue was dependent upon the presence of pyoverdine. While initially characterizing the Liquid-Killing assay, we discovered that several pathogenic mechanisms are employed by *P. aeruginosa* to kill *C. elegans* in liquid [15]. Pyoverdine-deficient mutants, such as those with deletions or transposon-insertion mutations, will still kill worms, but death occurs only after longer periods of exposure [15]. We took advantage of this phenomenon to ensure that PQ3 and PQ3c rescue through their effects on pyoverdine. The pyoverdine-deficient mutant strains PA14*pvdF* or PA14*pvdP* were used in Liquid-Killing assays in place of wild-type, isogenic *P. aeruginosa* strain PA14. Worms were treated with 5-FC, PQ3, PQ3c, or DMSO, and death was assessed at the same time-point as for wild-type *P. aeruginosa* (compare Figure 2c,d) or after the onset of pyoverdine-independent killing (Figure 2e). Under these conditions, neither 5-FC nor PQ3c showed significant rescue compared to DMSO (Figure 2d-e), suggesting that they are acting solely by preventing pyoverdine-mediated killing.

Next, we tested the ability of the pyoverdine inhibitors to limit bacterial growth and expression *pvdS* regulated genes [20,21,23,50]. The activity of PvdS is normally restricted by FpvR-mediated sequestration, which requires ferripyoverdine to bind its receptor FpvA to release the PvdS sigma factor [20,21,23,50]. While bacterial growth remained unchanged, pyoverdine production and expression of *pvdA, pvdE, pvdF, toxA*, and *prpL* decreased (Fig S2A-B). At the same type no decrease in the expression of pyoverdine-independent genes *pchD* and *pchR* was detected (Fig S2B). This indicates that compound binding is preventing pyoverdine-mediated gene regulation, a key function of this virulence factor.

**Figure 3.**
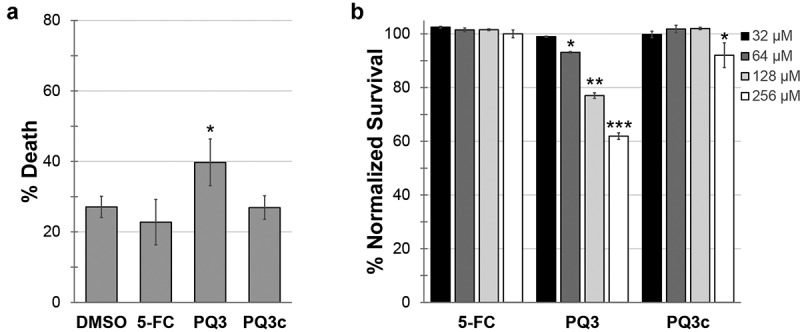
Toxicity of pyoverdine inhibitors to *C. elegans* and human cells. (a) Long-term (10 days) *C. elegans* survival in the presence of PQ3, PQ3c, 5-FC, or DMSO. (b) 16HBE cell survival was measure using alamarBlue dye after 24 h exposure to PQ3, PQ3c, 5-FC, or DMSO at specified concentration. All values are normalized to DMSO controls. At least three independent biological replicates were performed. For each replicate, at least four wells with ~20 worms/well (a) or at least 6 wells with cells at ~90% confluency (b). Statistical significance was determined using Student’s *t*-test.

Next, we substituted the *P. aeruginosa* in the Liquid-Killing assay with *E. coli* to test whether the compounds exhibit toxicity to *C. elegans* (Figure 3a). 5-FC and PQ3c were again indistinguishable from DMSO controls, suggesting that this concentration of compound is not harmful for *C. elegans*. In contrast, PQ3 exhibited both a slight toxicity and it also showed a weak but statistically significant ability to rescue against pyoverdine-independent pathogenesis (Figure 2d-e). These phenomena may be linked, as there are a number of previously published reports of molecules (RPW-24, platinum compounds, DMAQ-B1) that have short-term immunostimulatory benefits but long-term toxicity [51,52]. Compound toxicity was also evaluated in 16HBE cells, a human bronchial epithelial cell line (Figure 3b). Although significant dose-dependent cell death was observed for PQ3 treatment, this was almost entirely absent from the derivative molecule. Incubation with PQ3c resulted in less than 10% of cell death even at the highest concentration tested (256 µM).

### Generation of a conformational ensemble of apo-pyoverdine using molecular dynamics

In drug discovery, prior knowledge of the structure and dynamics of the biological target is required for ligand binding site determination and binding mode prediction. Previous NMR studies showed that pyoverdine is highly dynamic and adopts multiple conformations in solution at room temperature, which complicates characterization of the binding either by experimental structure tools or docking [53,54]. Therefore, to overcome this challenge, we applied MD simulation to generate a conformational ensemble of apo-pyoverdine by the selection of a representative set of snapshots that covers the expected range of flexibility and used them as targets for docking.

To develop mechanistic understanding of pyoverdine-ligand interactions, we started with investigation of the conformational dynamics of apo-pyoverdine by performing a 50 ns MD simulation using GROMACS software with a CHARMM27 force field [48]. The initial structure of pyoverdine was taken from the crystal structure of the FpvA-apo-pyoverdine complex (PDB ID: 1xkh) [49]. After initial solvation, energy minimization, equilibration, and production, the resultant trajectories were analyzed using the GROMACS analysis tool kit.

**Figure 4.**
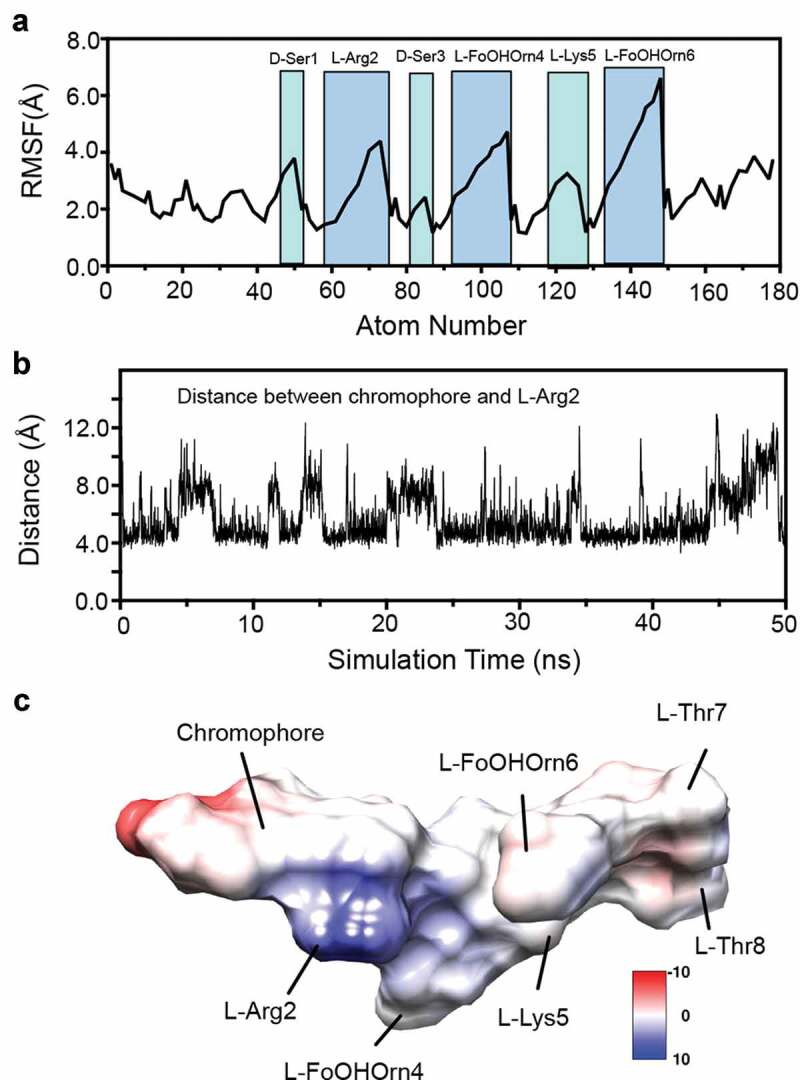
Conformational dynamics of apo-pyoverdine by a 50 ns MD simulation. (a) Root-mean-square fluctuation (RMSF) of backbone and sidechain heavy atoms in pyoverdine. Highlighted peaks correspond to labeled residues. (b) The center-to-center distances between the chromophore aromatic ring and L-Arg guanidinium group during the simulation. (c) Surface representation of the representative structure from Cluster 1 colored by Coulombic surface potential.

Analysis of MD trajectories suggested that apo-pyoverdine is very dynamic, as shown by high root-mean-square fluctuation (RMSF) values of the heavy atoms (Figure 4a). This is consistent with its relatively small size and high solubility in water. The large variations of RMSF suggested significant side-chain fluctuations for L-FoOHOrn4 (atom numbers 94–107) and L-FoOHOrn6 (atom numbers 140–149), whereas the sidechains of D-Ser1 and L-Arg2 showed less dynamic movements. Next, trajectories were grouped into root-mean-square deviation (RMSD)-based clusters using 2.5 Å cutoffs. Representative structures (Fig S3) were obtained by taking the median structure from each of the four most populated clusters.

As shown, the most common arrangement stacks the positively-charged sidechain of L-Arg2 underneath the aromatic chromophore, with the negatively-charged sidechain of L-FoOHOrn4 in the same or a similar orientation. The macrocycle, comprised of the four final amino acids (L-Lys5 through L-Thr8) at the C terminus, appears to be highly dynamic, which is consistent with the large changes in RMSF shown. This configuration gives rise to a shallow groove that is a potential ligand-binding site that has a high degree of conformational plasticity, as shown by the substantial variation in the center-to-center distances between the chromophore aromatic ring and the guanidinium group from L-Arg2 (Figure 4b). This is also shown in the coulomb surface model of the representative structure from Cluster 1 (Figure 4c) which accounts for the 56% of all MD models.

### Identification of PQ3 family binding sites by solution NMR

One straightforward and powerful method to prove and map the interactions between pyoverdine and the PQ3-family of pyoverdine inhibitors is to use solution NMR. Upon binding the compound, pyoverdine undergoes a conformational change that can be detected using heteronuclear single quantum correlation (HSQC) spectroscopy. In brief, this 2D NMR technique relies on the transfer of the magnetization from the proton directly attached to a heteronuclear atom, such as ^15^N or ^13^C, and back to proton [55]. The HSQC spectrum displays NMR resonances (peaks) or correlations that reflect the equivalent of a “frequency fingerprint” of the local chemical environment of these nuclei in the molecule. If the chemical environment changes, the NMR resonances will also change.

To obtain site-specific binding information by NMR, we first assigned ^1^H-^15^N HSQC spectra for apo-pyoverdine at 278 K and 298 K. The appearance of downfield ^1^H resonances at ~10 ppm at 278 K indicated lower temperature contributes to the stabilization of the conformational dynamics. As a result, all the NMR spectra in the present study were carried out at 278 K. Backbone and sidechain assignments for apo-pyoverdine were made using a series of triple resonance experiments (Fig S4). The assigned ^1^H-^15^N HSQC spectra of apo-pyoverdine at 278 K and 298 K are shown in Fig S5.

**Figure 5.**
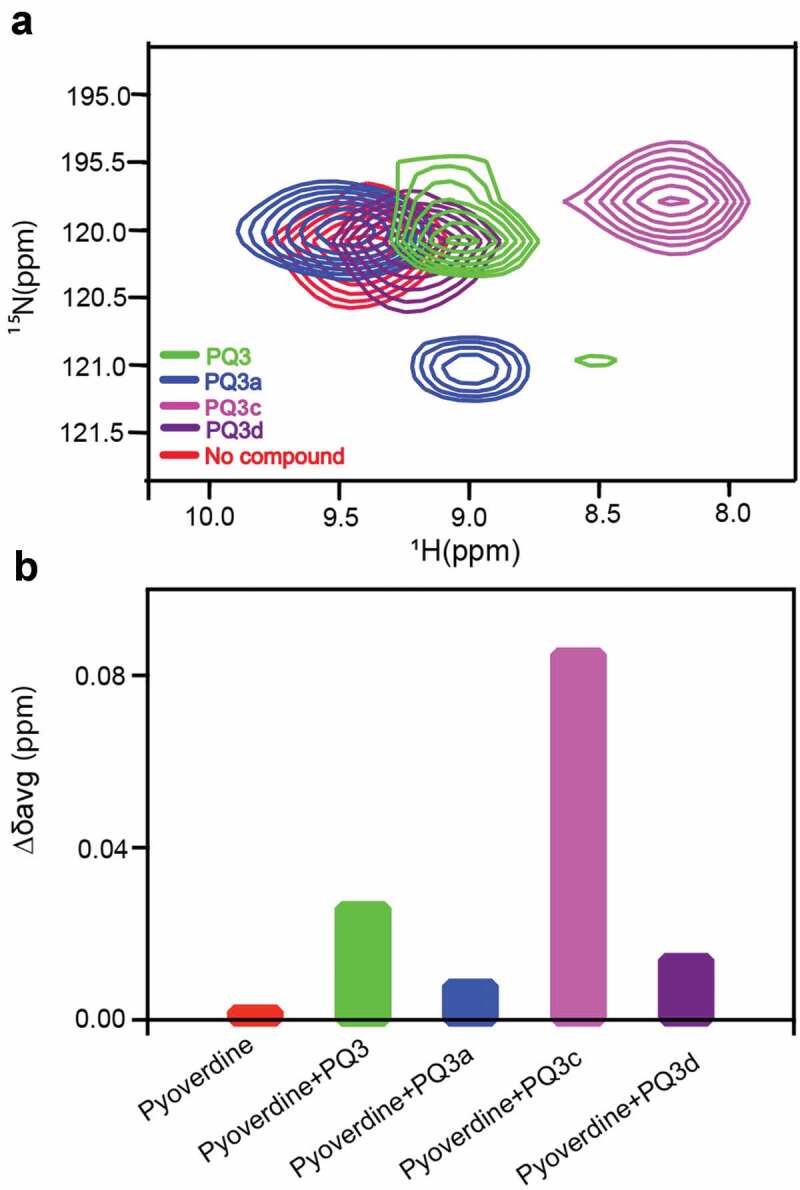
Effects of the PQ3-family compounds on ^1^H-^15^N HSQC spectrum of apo-pyoverdine. (a) N-terminal succinamide as probe to determine the positions of amide cross‐peaks from succinamide in the absence and presence of PQ3-family compounds. (b) Comparison of average amide chemical shift changes (Δδ_avg_) of succinamide in the absence and presence of compounds. Δδ_avg_ was calculated using Equation (2) in the Methods and Materials. Isotope-labeled pyoverdine concentration was 0.2 mM. The final concentration of each compound in the NMR sample was 2.5 mM with 5% (v/v) d-DMSO.

Next, ^1^H-^15^N HSQC was used to monitor the amide chemical shift perturbations to determine if PQ3 family compounds interact with pyoverdine. Each of the compounds that showed the ability to quench pyoverdine fluorescence (PQ3, PQ3a, PQ3c, and PQ3d), also caused amide chemical shift perturbations for the N-terminal succinamide, D-Ser1, L-Arg2, and D-Ser3 as well as Hε-Nε from L-Arg2 side chain (Figure 5a). In contrast, the amides from the pyoverdine macrocycle did not exhibit chemical shift changes (Fig S6). These results indicated that all of the compounds bind at or near the chromophore and the beginning of the oligopeptide, which is not surprising given their chemical similarity. We calculated the average amide chemical shift changes (Δδ_avg_) and compared these values for the N-terminal succinamide. This analysis showed that PQ3c exhibits the greatest Δδ_avg_ followed by PQ3, whereas PQ3d and PQ3a had much less effect on Δδ_avg_ (Figure 5b). Interestingly, the NMR chemical shift perturbations were consistent with the observations from fluorescence quenching assay.

**Figure 6.**
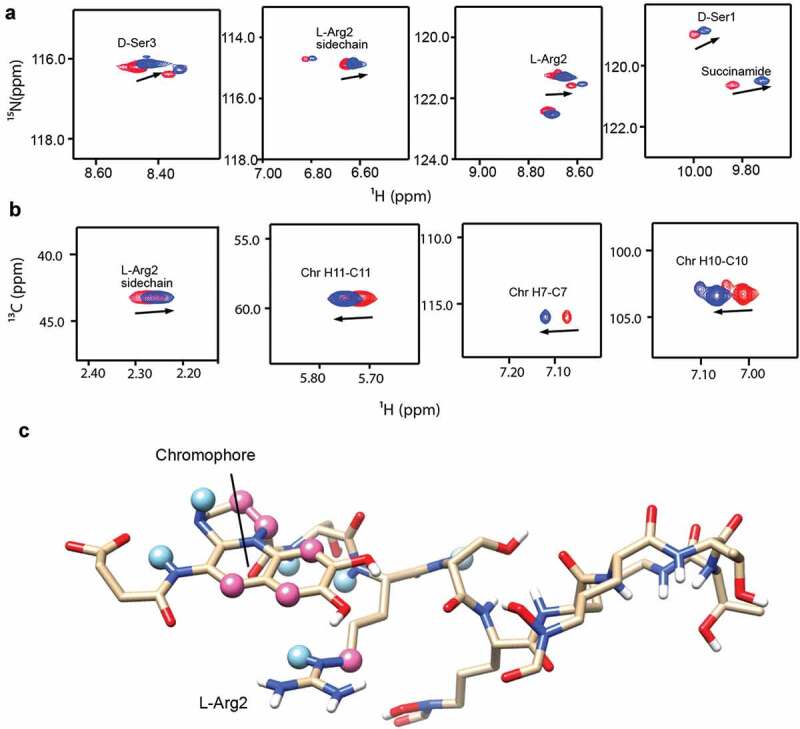
Characterization of the interactions between apo-pyoverdine and PQ3c. (a-b) NMR experiments were carried out at 278 K. The selected overlaid ^1^H-^15^N HSQC (a) or ^1^H-^13^C HSQC (b) spectra regions of pyoverdine in the absence (red) or presence of PQ3c (blue) are shown. (c) Pyoverdine residues, displaying induced chemical shift perturbations in the presence of PQ3c are labeled on the major conformation of pyoverdine (the representative structure of Cluster 1 from apo-pyoverdine MD simulation was used). Light blue spheres mark the atoms showing induced chemical shift changes in ^1^H-^15^N HSQC spectrum, pink spheres represent the atoms involved in the changes in ^1^H-^13^C HSQC spectrum. Isotope-labeled pyoverdine concentrations were used at 0.2 mM. The atom numbers used in (B) are based on the chemical structure of pyoverdine shown in Figure 1A.

Based on its properties, we selected PQ3c to map the binding site on pyoverdine. Of the analogs tested, PQ3c is unique in that it is highly soluble and non-toxic. It also displayed the best fluorescence quenching and was found to provide the greatest specific rescue in *C. elegans*. Additionally, PQ3c induces large chemical shift changes in pyoverdine. We used both ^1^H-^15^N HSQC and ^1^H-^13^C HSQC to monitor the amide chemical shift changes of the succinamide sidechain, D-Ser1, L-Arg2, and D-Ser3 (Figure 6a). This technique was also used to monitor the chromophore ring, and peptide sidechain (Figure 6b). Mapping the NMR spectral perturbations on the major conformation of pyoverdine indicated that the binding site for PQ3c largely overlaps with the location of the N-terminal groove revealed by MD simulation (Figure 6c).

### Docking of PQ3c to apo-pyoverdine

**Figure 7.**
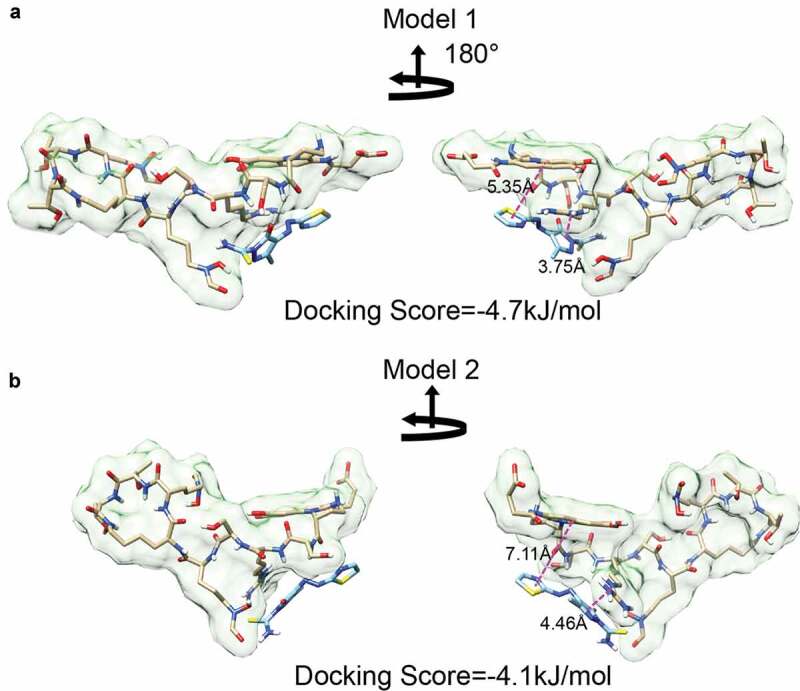
Molecular docking of PQ3c to apo-pyoverdine by AutoDock Vina. (a-b) Model 1 and 2, with the lowest docking scores in the docking experiment, were derived by using representative structures of Cluster 1 and Custer 2 as the starting structures, respectively. The center-to-center distances between the pyrazole ring and Arg guanidinium group and the distances between thiazole ring and chromophore aromatic ring are indicated.

Next, we used Autodock Vina to predict docking [44,45]. For this, we used the two median structures from the MD simulations as target structures with a rigid docking algorithm. The search space was limited to the PQ3c docking region identified by NMR. The resulting poses were then ranked by docking scores. For each target, PQ3c exhibited a similar conformation near the previously described shallow groove. Specifically, the pyrazole ring, with its negative charge, faced the positively-charged guanidinium group of L-Arg2, while the thiazole ring stacked with the aromatic ring of the chromophore, probably due to the delocalized electrons that are present in the conjugated systems of each molecule. Although the thiazole ring is shown at 180° orientations, the bond between the azo and thiazole groups rotates freely, making it likely that either pose can be readily found in solution. PQ3c appears to be associated more closely with apo-pyoverdine in model 1. This is shown by the smaller center-to-center distances between the pyrazole ring on PQ3c and L-Arg2 guanidinium group (3.75 Å in model 1 and 4.46 Å in model 2) and the distances between the thiazole ring on PQ3c and chromophore aromatic ring (5.35 Å in model 1 and 7.11 Å in model 2) (Figure 7a-b). Analogous experiments performed with the parental molecule, PQ3, demonstrated that it binds in a similar location but with slightly different interactions (Fig S7).

### MD simulation of PQ3c docking on apo-pyoverdine

**Figure 8.**
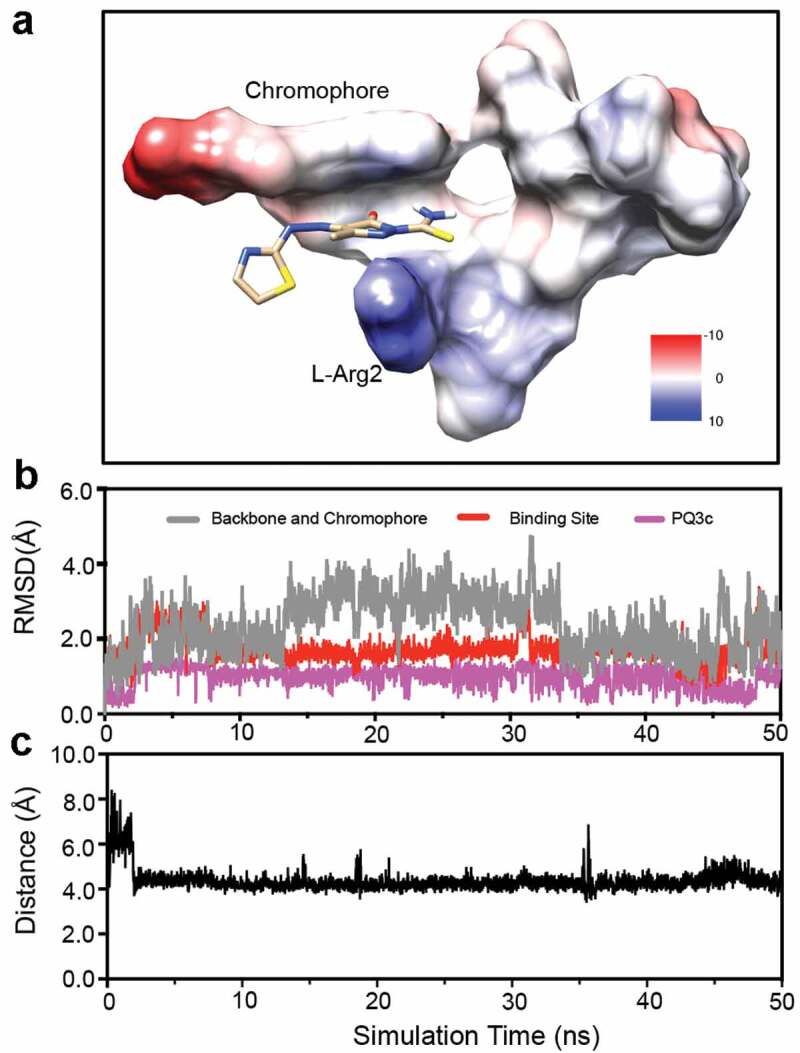
Molecular dynamics simulation of apo-pyoverdine – PQ3c complex. (a) Coulombic surface representation of the trajectory at the end of 50 ns MD simulation is shown. (b) Root-mean-square deviations (RMSDs) for chromophore and peptide backbone of pyoverdine, ligand binding site which includes the chromophore, D-Ser1 and L-Arg2, and PQ3c are calculated. (c) Calculated center-to-center distances between pyrazole ring on PQ3c and Arg guanidinium group in pyoverdine during the simulation are shown.

As targets were rigid during the docking search, the docking landscape could not properly account for the flexibility observed for pyoverdine. Therefore, the complex of model 1 and its docking with PQ3c was selected for the initial complex structure for optimization in a 50 ns MD simulation, which can account for solvent effects, conformational dynamics, and potentially correcting deficiencies associated with the docking protocol or scoring function. PQ3c, with a single negative charge, fits in the N-terminal groove, with the pyrazole moiety in close proximity to the guanidinium group of L-Arg2, as shown by the coulombic surface representation of the complex (Figure 8a).

The RMSD fluctuations were calculated for the heavy atoms of the chromophore and peptide backbone of pyoverdine, the small-molecule binding site (chromophore, D-Ser1and L-Arg2), and PQ3c to monitor dynamic stability. The RMSD values of PQ3c were 0.99 ± 0.28 Å, indicating PQ3c was stable at the binding site (Figure 8b). Compared to the peptide backbone, the PQ3c binding site on pyoverdine exhibited less conformational fluctuations, suggesting that PQ3c binding stabilizes the local conformation. The center-to-center distances between the pyrazole ring on PQ3c and the guanidinium group in L-Arg2 during the simulation were calculated to be 4.3 ± 0.5 Å (Figure 8c).

To visualize the interactions between PQ3c and particular regions of apo-pyoverdine in the binding site, a LIGPLOT [56] representation was generated (Fig S8). It showed that PQ3c occupied at N-terminal portion of pyoverdine; chromophore and D-Ser1 exhibited hydrophobic contacts with pyrazole and azo groups on PQ3c; the primary amine in the guanidinium group of L-Arg 2 formed a salt bridge with the pyrazole ring on PQ3c.

To assess the contributions of the individual residues to the overall binding of PQ3c, two types of short-range energies, Lennard–Jones short-range (LJ-SR) and Coulombic short-range (Coul-SR), were calculated to determine the interaction energy of PQ3c with the nearby pyoverdine residues (within 4 Å) throughout the MD trajectories (Table 2). Total LJ-SR and Coul-SR energies for 50 ns simulation were −75.01 kJ/mol and −148.19 kJ/mol, respectively, suggesting that Coulomb interaction contributes significantly to PQ3c binding. L-Arg2 showed a strong Coulombic interaction energy of −119.56 kJ/mol, indicating its crucial role in the electrostatic interaction. The aromatic chromophore is also involved in the binding energy, providing π-stacking interactions that generate substantial stability (−35.79 kJ/mol LJ-SR).

**Table 2.** Residue interaction energy analysis of pyoverdine-PQ3c complex for 50 ns MD simulation.

Residue	LJ-SR kJ/mol	Coul-SR kJ/mol
Chromophore	−35.79	−27.01
D-Ser 1	−7.0	−1.34
L-Arg 2	−19.49	−119.56
D-Ser 3	−7.37	0.23
Total	−75.01	−148.19

## Discussion

**Figure 9.**
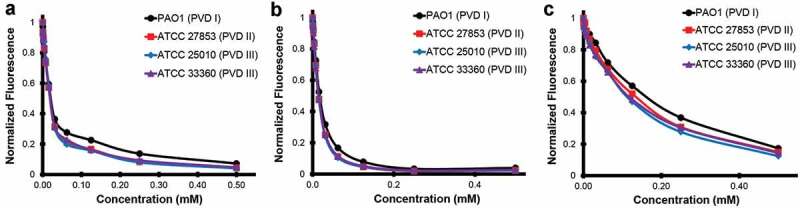
Pyoverdine inhibitors interact with multiple types of pyoverdine. Semi-purified pyoverdine from cultures producing each of the three major types of pyoverdine were incubated with pyoverdine inhibitors PQ3 (a), PQ3c (b), and the previously published inhibitor LK11 (c) at specified concentrations. Pyoverdine quenching was measured using its characteristic excitation/emission spectrum.

In this study we used a high-throughput chemical screen to identify small molecules that inhibit the fluorescence of the *P. aeruginosa* siderophore pyoverdine. Although a handful of hits were identified, only one hit, PQ3, showed sufficient activity to warrant follow-up. Taking advantage of commercial sources, we identified several analogs of PQ3. The best of these, PQ3c, shows both greater affinity and activity than PQ3. Using NMR in conjunction with MD simulations to structurally characterize the binding between PQ3 and pyoverdine, we identified an interaction site. The site includes contacts between the compound and regions of the chromophore and D-Ser1 and L-Arg2 of the oligopeptide. In particular, the negatively-charged pyrazole ring and the positively-charged ε-amine from L-Arg2 of the oligopeptide form a strong electrostatic interaction. Additional attractive nonpolar interactions, from π-stacking of the delocalized electrons in the semi-conjugated rings of the chromophore and the thiazole ring can also take place. Because the chromophore is shared by all fluorescent Pseudomonads and the 2nd amino acid in the peptide chain is a highly conserved residue (typically Arg or Lys [11]), we speculate that this binding site is likely to be conserved in pyoverdine from many strains, a supposition supported by the fact that representatives of all three types of pyoverdine are quenched by PQ3c and PQ3 (Figure 9). Each of the compounds was more effective at quenching fluorescence for all three pyoverdine types than a previously described compound, LK11 [9].

Results from our *in vivo* and *in vitro* assays contribute to a long-standing correlation between the ability to quench pyoverdine fluorescence and the ability to prevent pyoverdine functions (including binding iron and pathogenesis). In addition, our SAR suggests that compound's binding affinity correlates well with its ability to quench pyoverdine, which is consistent with the binding site near the chromophore. In this case, apo-pyoverdine’s affinity for PQ3c appears to depend on the pyrazole-1-carbothioamide moiety and the ring stacking with the chromophore. Although the small number of analogs tested and the relatively low affinity of the compounds limit the power of this SAR, it does suggest a few avenues for future developments.

PQ3c is an attractive scaffold for potential lead development due to its low molecular weight and single positive charge, which afford ample room to make modifications to enhance binding affinity. MD simulation of apo-pyoverdine indicated that the binding site has a high degree of plasticity, which might allow it to accommodate substitutions to the pyrazole ring, particularly if they are less bulky than the phenyl ring on the parent compound and less charged than the nitrosophenyl group on PQ3a. One possible strategy is to optimize the electrostatic interactions of this scaffold. For example, the introduction of a functional group on the thiazole ring that could contribute to an electrostatic interaction with the keto acid may lead to increased binding affinity. Another option would be replacement of the carbonothioyl functional group with a group that could form hydrogen bonds or hydrophobic contacts with the substituents on the pyrazole ring. Finally, replacement of the C-3 methyl group with an isopropyl group might also increase hydrophobic stabilization. As PQ3 and its derivative PQ3c still have relatively low affinity to pyoverdine, our efforts represent only a first step toward developing a lead compound.

Previous detailed studies of the interactions of pyoverdine with its receptor FpvA using fluorescence resonance energy transfer (FRET) have shown that FpvA binds both apo-pyoverdine and ferri-pyoverdine [57]. The three-dimensional structure of FpvA complexed with apo-pyoverdine has also been reported [49]. This structure shows a pocket where the siderophore interacts with the top of the β-barrel of FpvA. In these interactions, the sidechain of L-Arg2 reorients to form a salt bridge with the carboxylate group (COO-) of Asp597 in FpvA, and Tyr200, Arg204, and Tyr796 of FpvA interact with the chromophore of pyoverdine. Interestingly, Tyr200 in FpvA shows a similar π-stacking arrangement with the chromophore aromatic ring in pyoverdine (Fig S9A-C). Using the chromophore as a reference, superposition of free apo-pyoverdine and compound-bound pyoverdine illustrates that their peptide chains exhibit distinct conformational differences (Fig S9D). Based on our data, interactions between the compound and pyoverdine severely contort pyoverdine, and are likely to preclude interaction between pyoverdine and the FpvA receptor.

Ultimately, the question of whether anti-virulents will be a viable clinical tool, whether alone or in combination with conventional antibiotics, is building in importance. We present evidence here that PQ3c binds pyoverdine and that this decreases the production of pyoverdine-regulated virulence factors, likely by disrupting iron-binding or interactions with FpvA. These compounds clearly preclude normal siderophore function and, more importantly, limit its ability to inflict damage. This report shows a road map for the identification and improvement of virulence inhibitors. Considering the pressing danger of antimicrobial resistance, it is difficult to overstate the importance of these small-molecule therapeutics.

## Supplementary Material

Supplemental Material
