# Supplementary material for: Identification and validation of a novel anti-virulent that binds to pyoverdine and inhibits its function
Source: Virulence. 2020 Sep 22;11(1):1293–309. doi: 10.1080/21505594.2020.1819144 (PMC7549923; doi:10.1080/21505594.2020.1819144)
Supplement: Supplemental Material [file KVIR_A_1819144_SM6708.docx]

**Supplemental Figure S1. The chemical structures of PQ1, PQ2, PQ3, PQ4, and PQ5.**


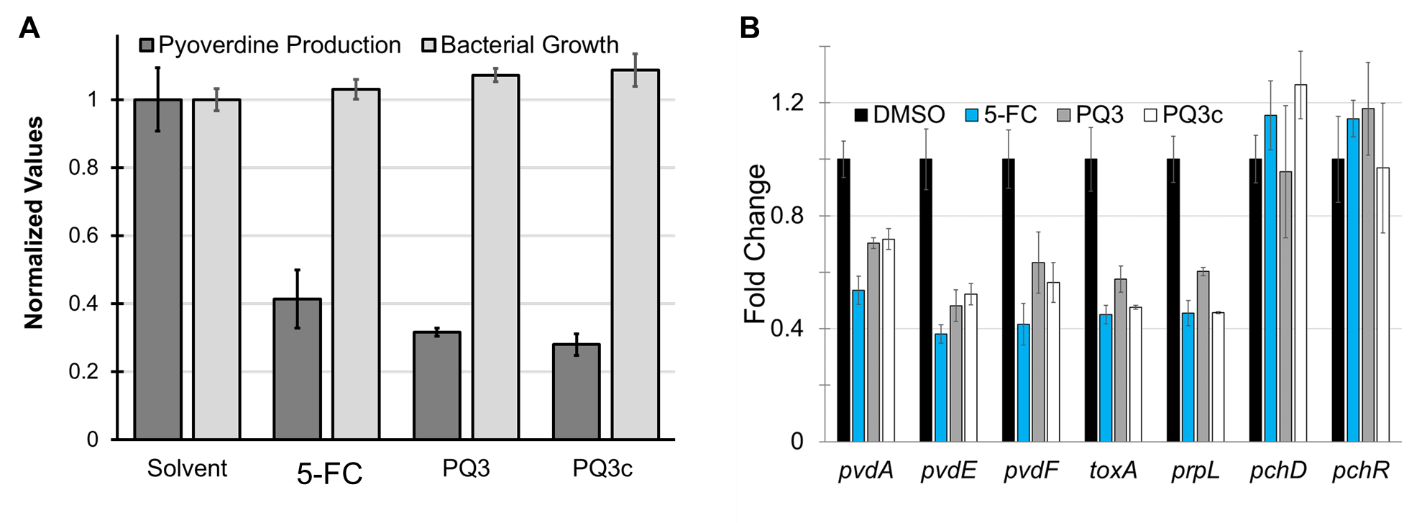


**Supplemental Figure S2. Pyoverdine inhibitors affect expression of pyoverdine-dependent genes, but not bacterial growth.** Bacteria growth or pyoverdine production (**A**) or expression of pyoverdine-dependent virulence genes (**B**) following *P. aeruginosa* growth in the presence of solvent control, or 100 µM of 5-FC, PQ3, or PQ3c.

**Supplemental Figure S3. Conformational ensemble of apo-pyoverdine derived from 50 ns MD simulation.** RMSD-based clustering with a cut-off of 2.5 Å were performed and the representative structures from the top four clusters are shown in sticks. The population of each cluster is also indicated.

**Supplemental Figure S4. Backbone assignments for apo-pyoverdine at 278K using 3D HNCACB and CBCA(CO)NH experiments.** The strip plots show the sequential assignment from L-Arg2 to L-Lys5. Pyoverdine sample for backbone assignments contained 1.2 mM ^13^C, ^15^N pyoverdine in 25 mM phosphate, 5% D2O, 5.0 µM 2,2‐dimethyl‐2‐silapentane‐5‐sulfonate (DSS), pH 5.8. The ^1^H, ^13^C, and ^15^N chemical shifts were deposited into Biological Magnetic Resonance Bank database (www.bmrb.wisc.edu) under the BMRB accession number 27960.

**Supplemental Figure S5. ^1^H**-**, ^15^N HSQC spectra of apo-pyoverdine produced by *P. aeruginosa* PAO1.** NMR spectra were collected at 278 K (**A**) or 298 K (**B**) and residue-specific assignment were indicated. The appearance of downfield ^1^H resonances at ~10 ppm at 278 K indicated that lower temperature contributes to the stabilization of the conformational dynamics*.* NMR sample contained 0.2 mM ^15^N apo-pyoverdine in 25 mM phosphate, 5% D_2_O, DSS, pH 5.8.

**Supplemental Figure S6. Residues at the C-terminal of apo pyoverdine are not affected by PQ3c binding at 278K.** The selected overlaid ^1^H-^15^N HSQC spectra regions of pyoverdine in the absence (red) and presence of PQ3c (blue) show that amides for L-Lys, L-Thr7 and L-Thr8 in pyoverdine exhibit little amide chemical shift perturbations upon PQ3c binding.


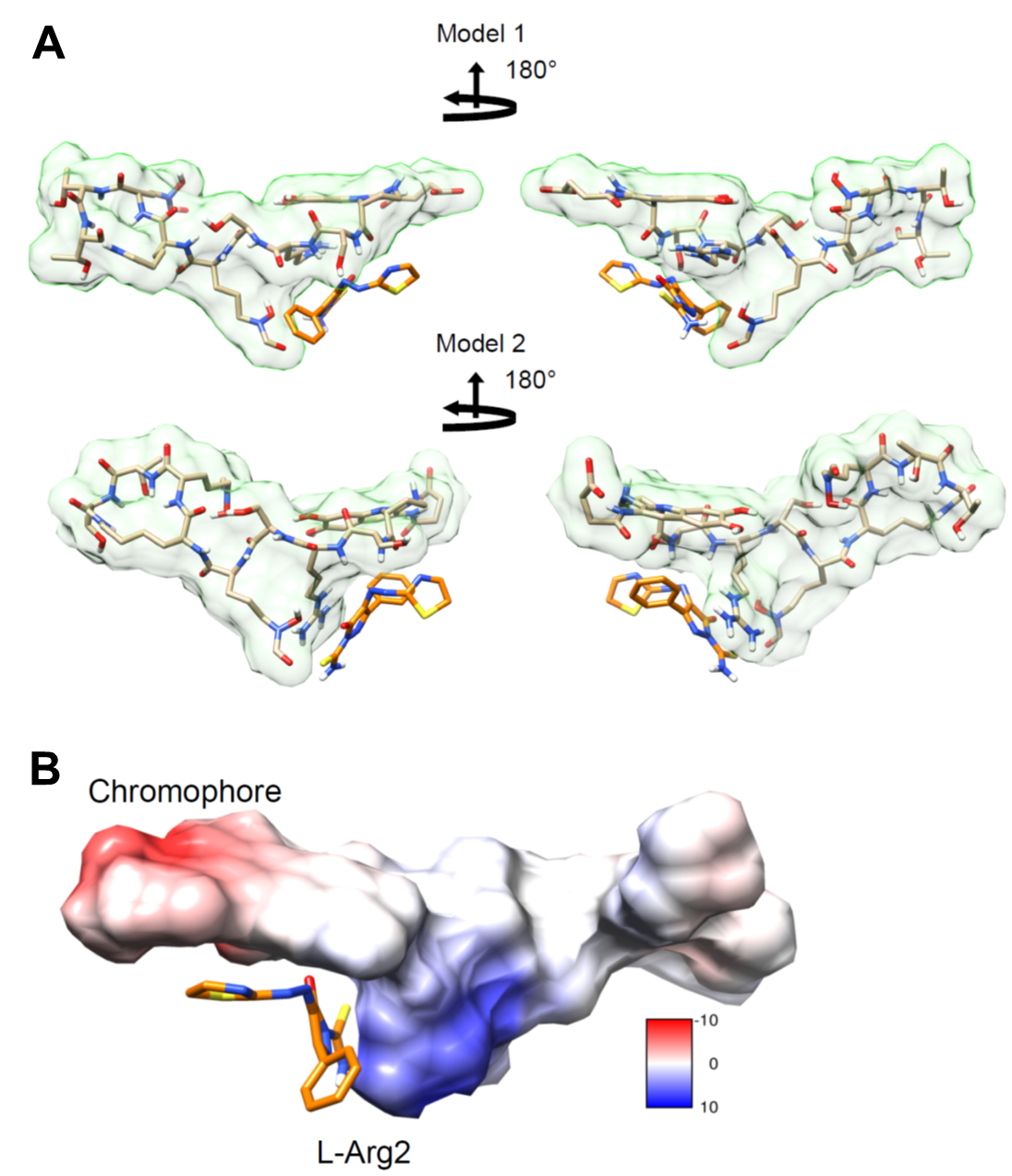


**Supplemental Figure S7. Molecular docking and molecular dynamics of PQ3 to apo-pyoverdine.** (**A**) Model 1 and 2, with the lowest docking scores in the docking experiment by AutoDock Vina, were derived using representative structures of **Cluster 1** and **Custer 2** as the starting structures, respectively. (**B**) Molecular dynamics simulation of the apo-pyoverdine – PQ3 complex using model 2 (**A**) as the starting structure. Coulombic surface representation of the trajectory at the end of 50 ns MD simulation is shown.

**Supplemental Figure S8**. **2D LIGPLOT representation of the interactions of apo-pyoverdine with PQ3c.** Chromophore and D-Ser 1 in pyoverdine involved in hydrophobic interactions are represented by brick red spoked arcs. L-Arg2 in pyoverdine involved in the electrostatic interaction is shown in green. PQ3c is shown in pink.

**Supplemental Figure S9. PQ3c binding has the potential to interfere the interactions between pyoverdine and its receptor FpvA**. (**A**) The X-ray crystal structure (PDB: 1XKH) of apo-pyoverdine bound to FpvA. FpvA and pyoverdine are shown in grey and pink, respectively. (**B**) As in (**A**) viewed down the axis of the β-barrel. (**C**) The interactions between apo-pyoverdine and neighboring residues (<3.6Å) in FpvA. (**D**) Superposition of apo-pyoverdine-PQ3c with apo-pyoverdine bound to FpvA. The chromophore is used as reference for structure superposition.
